# Advances and challenges in cell therapy for neuropathic pain based on mesenchymal stem cells

**DOI:** 10.3389/fcell.2025.1536566

**Published:** 2025-02-21

**Authors:** Wen-Jun Zhang, Xiong-Wei Pi, Dong-Xia Hu, Xiao-Ping Liu, Ming-Ming Wu

**Affiliations:** ^1^ Department of Rehabilitation Medicine, The Second Affiliated Hospital, Jiangxi Medical College, Nanchang University, Nanchang, Jiangxi, China; ^2^ The Second Affiliated Hospital, Jiangxi Medical College, Nanchang University, Nanchang, Jiangxi, China; ^3^ Department of Hepatobiliary Surgery, The Second Affiliated Hospital, Jiangxi Medical College, Nanchang University, Nanchang, Jiangxi, China

**Keywords:** mesenchymal stem cells (MSCs), neuropathic pain (NPP), nerve damage, treatment, translpantation

## Abstract

Neuropathic pain (NPP) is caused by damage to the somatosensory nervous system. Its prominent symptoms are spontaneous pain, hyperalgesia and abnormal pain. This pain is long-lasting and unbearable, seriously affecting the patient’s quality of life. At present, the clinical treatment effect of painkillers to relieve NPP is still not ideal, nor can it repair damaged nerves and achieve long-term treatment results. In recent years, the application of cell therapy strategies in the field of pain has yielded encouraging results, including preclinical studies and clinical trials. Mesenchymal stem cells (MSCs) are pluripotent progenitor cells derived from mesogenesis. They have the ability to self-renew and differentiate into multiple cell types and have been widely studied and applied in the field of neuroregenerative medicine. MSCs play an important mechanism functional role in promoting injured nerve regeneration and pain relief by regulating multiple processes in target cells, including immunoregulation, anti-inflammatory properties, promoting axon regeneration and re-myelination, promoting angiogenesis, and secreting neurotrophic factors. Moreover, MSCs can also release exosomes, which may be part of their analgesic effects. Exosomes derived from MSC also have the functional properties of mother cells and have therapeutic potential for treating NPP by promoting cell proliferation, regulating inflammatory responses, reducing cell death, promoting axon regeneration and angiogenesis. Therefore, in this article, we discussed current treatment strategies for NPP and explored the functional role and mechanism of MSCs in the treatment of NPP. We also analyzed the current problems and challenges in the application of MSCs in clinical trials of NPP.

## 1 Introduction

Direct or indirect damage to the nervous system can lead to dysfunction of the body, including sensory and motor functions, and induce pain. Pain is the body’s response to external injury or internal disease and is crucial to an individual’s risk perception and risk avoidance. Pain can be divided into acute pain and chronic pain according to time (defined as pain that persists or recedes for more than 3 months), while neuropathic pain (NPP) belongs to the type of chronic pain, caused by somatosensory nervous system lesions or diseases, accounting for 20%–25%, and its prevalence in the general population may be as high as 7%–8%, which has a significant impact on quality of life (Widerström-Noga, 2023; Bouhassira, 2019a). Most patients complain of persistent or intermittent spontaneous pain, especially light touches and cold. Pain may also spread to adjacent areas, leading to peripheral and central sensitization (Finnerup et al., 2021). The key to treat NPP lies in repairing damaged or stimulated sensory nerves, rebuild neural functional networks, and reduce sensory nerve transmission and synaptic plasticity. Although painkillers are often used clinically to relieve NPP, there are large individual differences in NPP, and the treatment effect is still not ideal. Therefore, exploring a promising treatment method seems very important.

Recently, with the continuous exploration of pain treatment methods, researchers have introduced cell replacement therapy strategies to transplant functional bioactive cells into the host to exert analgesic pharmacological effects (Deng et al., 2024). Compared with other methods, such as drug therapy or substance therapy, cell transplantation has key advantages in repairing damaged nerves, providing a good environment for nerve regeneration, and rebuilding neural network function. MSCs are multi-lineage cells with self-renewal and differentiation potential and play a key role in tissue healing and regenerative medicine. MSCs have homing capabilities, which means they can migrate to the injured site and have the ability to differentiate into local components of the injured site, as well as the ability to secrete chemokines, cytokines, and growth factors that help tissue regeneration (Fu et al., 2019; Li and Jiang, 2011). MSCs can also move into the peripheral circulation and pass through the blood vessel wall to reach target tissues, and repair damaged nerves. MSCs are the most commonly used stem cells in the treatment of nerve injury and pain. Their analgesic functions include promoting axon regeneration, re-myelination, immunomodulation and anti-inflammation, protecting neuron survival, and promoting blood vessel formation (Zaripova et al., 2023; Bryk et al., 2022). Studies have shown that intrathecal injection of bone marrow MSCs targeting the TLR2/MyD88/NF-kB pathway in spinal dorsal horn microglia triggers neuroprotection and sustained NPP relief through TSG-6 secretion (Bryk et al., 2022; Yang et al., 2020a). In addition, exosomes produced by MSCs have been shown to relieve pain symptoms with fewer side effects, and have potential application value in pain treatment applications (Ren et al., 2019a). MSCs have strong paracrine ability, and exosomes, as the main secretory group of MSCs, are considered to be important regulatory mediators involved in nerve regeneration. MSC-derived exosomes act as small transporters that can easily cross the blood-brain barrier, which has given rise to great interest in treating various neurodegenerative diseases, including pain (Bhujel et al., 2022). As nanocarriers, exosomes can transport various endogenous or foreign bioactive substances to recipient cells, promote blood vessel and axon regeneration, promote cell proliferation, inhibit cell death and anti-inflammation, thereby inhibiting the functional role of pain progression (Li et al., 2024; Liu et al. 2021a). Studies have shown that exosomes derived from adipose tissue MSCs encapsulated in collagen and fibrin hydrogel scaffolds can promote damaged nerve repair and reduce NPP in rats with spinal cord injury (Afsartala et al., 2023). These studies have revealed that MSCs can serve as therapeutic target cells for pain treatment. Therefore, we focused on the functional role of MSCs in the therapeutic application of NPP and the current problems and challenges in clinical trials.

## 2 Neuropathic pain and its current treatment status

NPP is a common symptom experienced by most diseases, including multiple peripheral or central diseases (Jensen et al., 2011). This pain is usually observed in the innervation areas of the body where nervous system structures are damaged (projected pain) (Treede et al., 2019). In 2019, the International Association for the Study of Pain (IASP) classified NPP in detail, dividing it into chronic peripheral NPP and chronic central NPP (Scholz et al., 2019). The new classification lists the most common chronic peripheral NPP caused by lesions or diseases of the peripheral somatosensory nervous system, mainly including trigeminal neuralgia, peripheral nerve injury, painful polyneuropathy, postherpetic neuralgia and painful radiculopathy. Chronic central NPP is caused by central nervous system injury or disease, including chronic central NPP associated with spinal cord injury, chronic central NPP associated with brain injury, chronic post-stroke pain, chronic central NPP caused by multiple sclerosis and other specific and unspecified chronic central NPP (Scholz et al., 2019).

The sources of NPP are diverse, such as trauma, pressure, immunity, tumor invasion and inflammation. These factors can cause varying degrees of nerve damage and induce paresthesia changes. Clinically, NPP is characterized by a combination of positive and negative phenomena. Positive phenomena include various pain symptoms, paresthesia and/or sensory disorders, which by definition are abnormal non-pain sensations (such as tingling, numbness, and acupuncture). Negative phenomena usually include neurosensory deficits in the pain area, as well as other deficits (motor, cognition, etc.), depending on the lesion site (Bouhassira, 2019b). Since NPP is essentially a subjective experience described by the patient’s specific symptoms. In addition, due to individual differences, age and gender differences, the use of damaged or stimulated nerves, and individual differences in sensitivity to pain, these can lead to clinical manifestations of pain. There are large differences in the form, which also brings huge challenges to clinical judgment, diagnosis and treatment. Current screening tools can only be expressed in the form of questionnaires, such as the NPP Questionnaire, PainDetect, ID-Pain and DN4, which classify NPP based on verbal descriptions of the quality of pain reported by patients (Baron et al., 2010). At the same time, a diagnosis of suspected NPP requires special examinations to determine whether the pain originates from the nervous system. The distribution of pain must correspond to potential damage or disease to the somatosensory nervous system (Baron et al., 2010). Electrophysiological techniques and neural biopsy samples can help assess the decline of neurological function and record the extent of neuropathy, but non-invasive diagnostic techniques still need to be explored.

At present, the treatment of NPP is still dominated by drug use. The first-line drugs for the treatment of NPP include gabapentin (gabapentin and pregabalin), bicyclic antidepressants (TCAS) and selective Licorine-norepinephrine reabsorption inhibitors (SNRI). Lidocaine, capsaicin and tramadol are recommended as second-line treatments, while powerful opioids (morphine and oxycodone) and botulinum toxin A (BTX-A) are listed as third-line treatments for peripheral NPP (Cavalli et al., 2019). These drugs have unsatisfactory analgesic effects in clinical applications, and can even achieve only slight effects. These drugs are central and often produce typical central side effects, such as sedation and dizziness. Bicyclic antidepressants also have significant anticholinergic and sedative side effects and a potential risk of falls. In addition, drugs can also lead to drug use dependence, resulting in reduced efficacy and truncation in long-term use. Therefore, innovative methods are needed to improve treatment strategies. These mainly include reasonable combination therapy, drug reuse, non-pharmaceutical methods (such as neurostimulation techniques), physical therapy, nerve blocks and personalized treatment management (Attal et al., 2023). However, these methods are still not ideal in terms of pain relief and cannot achieve long-term treatment results. Therefore, it is very important to explore a promising application treatment method.

## 3 A brief introduction to the biological characteristics of MSCs

MSCs, also known as mesenchymal matrix cells, have the characteristics of stem cells and have the characteristics of infinite proliferation and differentiation. Human MSCs exist in adult bone marrow and in various tissues such as adipose tissue, nerve tissue, cord blood and dermis, and can replicate and regenerate as undifferentiated cells. These cells showed a stable phenotype and retained their multi-directional differentiation potential *in vitro* (Pittenger et al., 1999; Galipeau and Sensébé, 2018). MSCs can be identified by expressing specific cell surface markers (CD105 +, CD73 +, CD90 +, CD34−, CD14−, CD11b−, CD79−, CD19−, or HLA-DR) (Uder et al., 2018a). MSCs can be induced to differentiate into mesenchymal lineages, such as osteoblasts, chondrocytes, adipocytes, endothelial cells, and cardiomyocytes, and non-mesenchymal lineages, such as hepatocytes and neuronal cell types.

Another important feature is that MSCs have low immunogenicity. MSCs does not express human leukocyte antigen (HLA) class II molecules and can hardly induce the proliferation of allogeneic lymphocytes (Le Blanc and Ringdén, 2007). In addition, bone marrow MSCs appear to have immunosuppressive effects *in vitro*. They inhibit the proliferation of T cells against alloantigens and mitogens, and prevent the development of cytotoxic T cells. However, MSCs can prolong the survival time of allogeneic skin grafts *in vivo* and have multiple immunomodulatory effects (Dominici et al., 2006). Moreover, MSCs have plastic adhesion, which is a characteristic that is different from other types of stem cells (Uder et al., 2018b). Their multifunctional biological qualities include immunomodulatory, anti-inflammatory and pro-regenerative capabilities, and rely largely on the migration and secretion capabilities of MSCs (Uder et al., 2018b).

Furthermore, MSCs have immunomodulatory and anti-inflammatory properties, express anti-inflammatory markers (such as IL-10, TSG6 and IDO) and immunomodulatory molecules (such as GF, VEGF-CXCR4), and regulate immune cell activities, which also provides a good basic microenvironment for tissue regeneration and nerve damage repair (Li et al., 2022; Hazrati et al., 2022). It is precisely because of their self-renewal ability, pluripotency and low immune characteristics that they have become the highlight of regenerative medicine cell therapy and are currently the main source of cells for the replacement of damaged tissues.

## 4 The advantages and mechanisms of MSCs-based analgesia therapy

As mentioned earlier, MSCs have the functions of multi-directional differentiation, promoting stem cell implantation, low immunogenicity and immunoregulation, and have now been proven to be an effective alternative to treating pain. MSCs exert their analgesic advantages and mechanisms, mainly reflected in the characteristics of promoting axon regeneration and myelination, secreting neurotrophic factors, promoting angiogenesis, immunoregulation and anti-inflammation, and providing a promising analgesic treatment method.

### 4.1 MSCs promote axon regeneration and re-myelination

The key to the neural injury repair process lies in axon regeneration and re-myelination, create new axon connections to form a neural network with the host, reduce nerve impulse conduction and synaptic plasticity, and facilitate pain relief. The first step in the regeneration process of nerve injury is to travel through the injured area to connect with the distant target nerve to form a new neural network (Zhang et al., 2023). Therefore, promoting axon regeneration in the injured area is the key to treat nerve injury regeneration and pain relief. Different studies have determined that MSCs can promote axon regeneration after nerve injury and can serve as a guide to target new axons to reach long distances and form new links. MSCs transplanted to the injury site can replace damaged tissue cells, promote axon regeneration and myelination, and play a functional role in nerve bridging (Gonen et al., 2024). MSCs facilitates axon regeneration and re-myelination of nerve fibers, promotes the increase of the number of myelinated fibers, thickness of myelin sheath, number of axons and expression of growth factors, and improves the recovery of motor function (Gonen et al., 2024; Ortiz et al., 2022a).

Ability of human placenta-derived MSCs to stimulate the regeneration of damaged rat retinal neurons by promoting axon growth and restoring neuronal activity under normoxic and hypoxic conditions (de Laorden et al., 2023a). MSCs promote the regeneration of 10%–14% of neurons and increase the axon length per neuron (de Laorden et al., 2023a). Umbilical cord derived MSCs significantly improve the decrease in sciatic nerve myelin basic protein levels and sciatic nerve demyelination caused by partial ligation of the sciatic nerve, inhibit neuron damage and promote myelin regeneration (Miyano et al., 2022). Other studies have shown that transplantation of umbilical cord derived MSCs to sciatic nerve injuries increases the number of axons regeneration, increases the density of neurofilaments, and improves sciatic nerve function (Ülger et al., 2021). These studies have revealed that MSCs are play a functional role in promoting axon regeneration in nerve regeneration.

**FIGURE 1:**
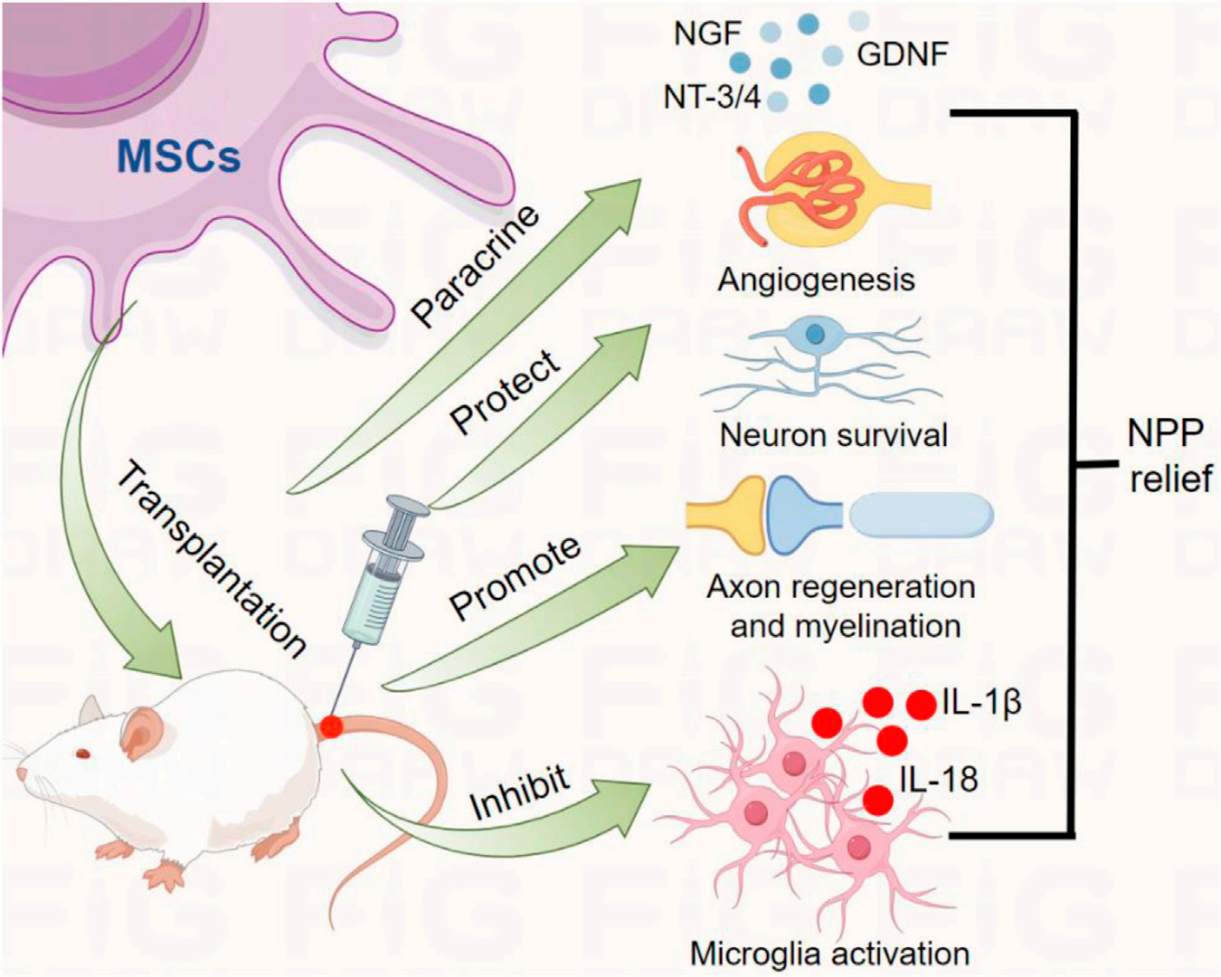
MSCs transplantation produces analgesic functional effects. Transplantation of MSCs into the body can produce analgesic functional effects by secreting neurotrophic factors (such as NGF, GDNF, and NT3/4), promoting blood vessel formation, promoting axon regeneration and myelination, inhibiting inflammatory cell activation and the release of pro-inflammatory cytokines.

MSCs can relieve NPP by promoting axon regeneration and improving nerve function (Ren et al., 2019b) (Figure 1). Amniotic fluid-derived MSCs can be recruited through the expression of SDF-1a in injured muscles and sciatic nerves. Increased deposition of amniotic fluid-derived MSCs alters the expression profile of SDF-1a in muscles or nerves, leads to significant improvements in neural behavior, electrophysiological function, and myelination in animal axons (Yang et al., 2012). The damaged sciatic nerve fibers in rats showed swelling, vacuolar degeneration of the myelin sheath, and unclear nerve fiber boundaries, while amniotic fluid-derived MSCs significantly increased the axial cylinder of the nerve and increased the myelin sheath of the sciatic nerve after transplantation (Yerofeyeva et al., 2023). Transplantation of fat-derived MSCs accelerates dynamic gait recovery in rats with sciatic nerve injury, eliminates static gait disorders, promotes nerve regeneration and relieves NPP (Yerofeyeva et al., 2023). Transplantation of MSCs into the spinal cord reduces the volume of spinal cord injury and protects the sensory related fibers of the μ-opioid receptor (MOR, interneuron) and serotonin transporter (HTT, descending pain inhibitory pathway) around the dorsal horn of the spinal cord, effectively promoting functional recovery and alleviating NPP (Yamazaki et al., 2021).

Transplantation of GABAergic neuron cells can relieve pain after spinal cord injury and improve motor function (Hwang et al., 2016). MSCs can differentiate into a sufficient number of GABAergic neuron cells to play an analgesic role. cAMP-responsive element-binding protein 1 (EREB1) is overexpressed in human mesenchymal stem cells (hMSCs), inducing differentiation into GABAergic neuron cells, enhancing GABAergic neural differentiation. Intrathecally transplanted hMSCs transfected with CREB1 into spinal cord injury showed the neuronal markers Tuj1 and Map2, improved neuronal survival and neural differentiation, increased thermal pain latency, and alleviated NPP (Kao et al., 2023). Transplantation of spinal cord derived MSCs and umbilical cord derived MSCs into spinal cord injury rats can protect neuron survival, improve functional recovery, and reduce allodynia and hyperalgesia, while umbilical cord derived MSCs have better therapeutic effect (Yousefifard et al., 2016). These studies reveal that MSCs can differentiate neurons, protect neuron survival, promote axon regeneration and myelination, thereby achieving analgesic functions. This also means that replacing axon lysis and demyelination and neuronal differentiation by promoting axon regeneration and protecting neuron survival is the key to alleviating NPP.

### 4.2 MSCs produce neurotrophic factors

Neurotrophic factors (NT) belong to a family of trophic factors that regulate neuron survival, growth and programmed cell death, including nerve growth factor (NGF), brain-derived neurotrophic factor (BDNF), and neurotrophin 3/4 (NT 3/4) (Khan and Smith, 2015). Neurotrophic factors promote the survival and growth of neurons *in vitro*. *In vivo*, neurons rely heavily on limited neurotrophic factors during development. After peripheral nerve injury, the adult mammalian peripheral nervous system responds to nerve regeneration by providing neurotrophic factors through autocrine or paracrine sources (Boyd and Gordon, 2003; Lien et al., 2020). Current models of NPP recognize the importance of ectopic activity of primary sensory neurons in the sensitized central nervous system. Neurotrophic factors have been shown to have neuroprotective effects on damaged sensory neurons, providing protection from their role in NPP states (Boucher and McMahon, 2001; Sah et al., 2003). Recent data suggest that a neurogenic factor (neurotrophic factor derived from a glial cell line) has a powerful analgesic effect in an animal model of NPP (Boucher and McMahon, 2001). Most of these neurofactors are involved in the underlying mechanisms of NPP through their respective associated high-affinity neurotrophic caseins (such as TrkA and TrkB receptors) signaling (Khan and Smith, 2015). For example, NGF binds to TrkA with high affinity, BDNF and NT-4 bind to TrkB with high affinity, and NT-3 bind to TrkC with high affinity. In addition to activating TrkC, NT-3 also activates TrkA and TrkB (Sumarwoto et al., 2021).

MSCs transplantation can provide a good basic nutritional environment for nerve injury regeneration and repair by paracrining multiple neurotrophic factors, such as VEGF, CNTF, NGF and BDNF (Sumarwoto et al., 2021; Chen et al., 2023) (Figure 1). These neurotrophic factors are expressed at high levels in MSCs (Pan et al., 2007). Human placenta-derived MSCs can participate in neuron regeneration and the recovery of neuron physiological activities by producing BDNF, NGF and NT-3 (de Laorden et al., 2023b). Tooth derived MSCs produce neurotrophic factors, including nerve growth factor, brain-derived neurotrophic factor, and glial cell line-derived neurotrophic factor, which promotes peripheral nerve regeneration and prevent facial motor neuron death (Sasaki et al., 2021). Bone marrow MSCs can relieve pain progression by secreting GDNF to inhibit the M1 phenotype of microglia and promote the M2 phenotype, inhibit NF-kB and PI3K/AKT signaling activation pathway (Zhong et al., 2020). Further research showed that MSCs combined with pregabalin significantly increased the expression of BDNF (reduced anti-inflammatory cytokines) in sciatic nerve injured rats, improved sciatic nerve structure and function, and alleviated NPP (Yousof et al., 2021). In addition, MSC-derived exosomes are rich in these nutritional factors, and produce analgesic pharmacological effects by transplanting stably expressed exosomes into the host. Exosomes derived from bone marrow MSCs can relieve heat pain allergies and mechanical allodynia in rats by transporting GDNF (Zhang et al., 2024).

It is understood that some protein receptors such as P2X purinergic receptors and TRPV1 can be involved in the occurrence and development of NPP as pain-related molecules (Rawish and Langer, 2022; Katz et al., 2023). These factors show a high expression pattern in the progression of pain. MSCs transplantation can play a functional role in alleviating NPP by down-regulating the expression of these pain-related factors. Studies have shown that MSCs-conditioned medium reduce mechanical allodynia and thermal hyperalgesia in NPP rats, and their anti-nociceptive effects are partly mediated by preventing the upregulation of P2X4 and P2X7 receptors in the spinal cord (Masoodifar et al., 2021). Implantation of human umbilical cord MSCs to encapsulate Gelfoam can improve motor dysfunction and inhibit NPP induced by spinal cord injury. Their analgesic function may be by promoting nerve regeneration, myelination and synapse formation (increased expression of NF200, MBE, GAP43, synaptophysin and PSD95) and reducing expression of pain-related proteins (TRPV1 and Cav3.2) (Poongodi et al., 2024). These studies reveal the fact that MSCs not only expresses multiple neurotrophic factors, lays a nutritional foundation for pain relief, but also produces analgesic effects by down-regulating the expression of pain-related factors.

### 4.3 MSCs promote angiogenesis

The vascular system promotes nerve development and neurogenesis by supplying tissues and cells with blood, oxygen and other nutrients, and maintains the stability of nervous system function (Liu et al., 2024; Li et al., 2021). Damage or occlusion of blood vessels can lead to ischemia and tissue necrosis, which is detrimental to tissue repair. The NPP process covers a variety of pathophysiological states. An important point is that ischemic injury, especially in the central nervous system, angiogenesis is significant in neuron survival and NPP (Sharma et al., 2023; Weng et al., 2024). Therefore, promoting angiogenesis restores function after nerve injury and alleviates NPP. MSCs transplantation can promote angiogenesis in the injured area and promote nerve injury repair (Ortiz et al., 2022b). Induction pluripotent stem cell-derived MSCs transplanted into a rat model of peripheral nerve injury survived for up to 17 days, promoted new blood vessel formation, accelerated the regeneration of damaged axons in rats and improved behavioral function (Meng et al., 2024).

MSCs, including their secretory exosomes, can play an important role in blood vessel formation and regeneration by regulating endothelial cell activity (Yin et al., 2016). Exosomes isolated and identified from human umbilical cord MSCs can promote endothelial cell proliferation, migration and angiogenesis in a dose-dependent manner (Yin et al., 2016). Human umbilical cord MSC-derived exosomes promote wound healing and angiogenesis, which may mediate Wnt4-induced β-catenin activation in endothelial cells and play a pro-angiogenic role (Zhang et al., 2015a). Exosomes derived from human umbilical cord MSCs contain angiopoietin-2, which promotes the proliferation, migration and blood vessel formation of vascular endothelial cells (Liu J. et al., 2021b). Transplantation of exosomes derived from pluripotent MSCs significantly increased the number of newly formed endothelial cells in the lesion boundary area and dentate gyrus of traumatic brain injury in rats, and increased the number of newly formed immature and mature neurons in dentate gyrus, and reduced neuroinflammation and effectively improved functional recovery (Zhang et al., 2015b). Further studies have shown that MSC-derived exosomes effectively improve functional recovery after cerebral hemorrhage, possibly by promoting endogenous angiogenesis and neurogenesis in rats after cerebral hemorrhage, including increasing mature neurons and myelination (Han et al., 2019). In a rat model of oxaliplatin-induced neuropathy, intravenous injection of adipose MSCs reduced oxaliplatin-dependent mechanical hypersensitivity, which took effect 1 h after dosing and peaked 6 h later for 5 days, increased plasma concentrations of VEGF in rats and upregulation of VEGF 165b in the spinal cord (Di Cesare Mannelli et al., 2018). These studies have confirmed the functional role of MSCs in angiogenesis and can participate in angiogenesis by regulating endothelial cell functions. Although there are few reports on the functional role of MSCs in promoting angiogenesis in pain relief and there is a lack of sufficient direct evidence, it is undeniable that MSCs participate in the functional role of angiogenesis and nerve regeneration.

### 4.4 MSCs immunoregulation and anti-inflammation

The inflammatory process mediates the activation of immune cells and the release of inflammatory mediators, such as pro-inflammatory cytokines and chemokines can induce pain by directly activating nociceptors (primary sensory neurons that detect harmful stimuli) (Matsuda et al., 2019; Jiang et al., 2023). The neuroinflammatory response mediated by immune cell activation leads to persistent myelin loss and axon damage, which is closely related to functional recovery and pain after nerve injury (Jiang et al., 2023). One feature of neuroinflammation is the activation of glial cells in the dorsal root ganglia, spinal cord and brain, leading to the production of pro-inflammatory cytokines and chemokines in the peripheral and central nervous systems, driving peripheral and central sensitization (Matsuda et al., 2019). Early and late inflammation may have a unique impact on the onset and relief of pain. Nerve injury causes glial and immune cells in the peripheral nervous system to be activated, releasing pro-inflammatory mediators, promoting sensitization of nociceptors, and causing pain. Neuroinflammation in the central nervous system drives central sensitization and promotes the development of chronic pain (Fang et al., 2023). Therefore, inhibited the release of inflammatory cytokines mediated by immune cell, reduced neuroinflammatory responses, improved the inflammatory microenvironment of nerve injury, and relieved NPP.

MSCs suppresses the immune response of innate immune cells, including macrophages, dendritic cells (DC), natural killer cells (NK cells), and natural killer T (NKT) cells, and adaptive immune cells, including T cells, B cells, and other liver-specific immune cells, to create a tolerant microenvironment (Chen et al., 2022; Hu and Li, 2019). MSCs immunomodulation can be achieved through cellular contact and/or secretion of multiple factors. Many studies believe that MSCs and immune cells establish a bidirectional regulatory mechanism; the activation of the immune regulatory properties of MSCs requires the presence of pro-inflammatory cytokines from immune cells (Castro-Manrreza and Montesinos, 2015; Jiang et al., 2021). Another biological characteristic of MSCs is their strong immunoregulatory and immunosuppressive properties, which can participate in the progression of NPP by secreting biologically active factors or regulating immune cell activation. One functional anti-inflammatory effect of MSCs is by reducing or down-regulating the expression of inflammatory cytokines such as CCL2, CCL5, CXCL10, TNF-α, interleukin-6 and IL-1β (Watanabe et al., 2015; Yang et al., 2016). Transplantation of MSCs spheroids into the injured sciatic muscles can more effectively relieve pain. These spheroids play an anti-inflammatory role by weakening the expression of mouse-derived genes Ccl11/Eotaxin, leukocyte factor 1A, TNF-β and tumor necrosis factor that are associated with the inflammatory response (Lee et al., 2022). Another study showed that high-dose bone marrow MSCs transplantation can relieve pain earlier, significantly reduce spinal IL-1β and TNF-a levels, and have stronger analgesic and anti-inflammatory effects than low-dose bone marrow MSCs transplantation (Sezer et al., 2022).

Microglia are resident glial cells in the central nervous system and play a key functional role in maintaining the immune microenvironment and functions of the nervous system. As nerve injury or pain progresses, microglia can be activated and migrate to the injury site to produce immune regulation. MSCs can produce anti-nociceptive effects by regulating microglia polarization and neuroinflammatory response (Figure 1). Transplantation of bone marrow MSCs into spinal cord tissue inhibits neuroinflammation induced by sciatic nerve injury, reduces the production of pro-inflammatory cytokines (IL-1β, IL-6 and TNF-a) in microglia, produces anti-inflammatory effects, provides neuroprotection and sustained NPP relief (Yang et al., 2020b). Intravenous injection of human umbilical cord MSCs alleviates NPP in rats by inhibiting microglia activation in the injured spinal cord dorsal horn and reducing sciatic nerve injury (Xu et al., 2024). Human umbilical cord MSCs improve mechanical allodynia and thermal hyperalgesia induced by spinal cord injury, which is associated with the inhibitory process of neuroinflammation, including inhibition of activated astrocytes and microglia, reduction of the pro-inflammatory cytokines IL-1β and IL-17A, and upregulation of the anti-cytokine leukocyte leukocyte leucocyte-10 (IL-10) (Chen et al., 2016).

In addition, in view of the immune and anti-inflammatory functions of MSCs in NPP, some studies have engineered MSCs to enhance their functions to produce better analgesic treatment effects (Yu et al., 2015). Compared with human MSCs transplantation, expression of sirtuin 1 (SIRT1) enhances the function of human MSCs, better reduces the level of pro-inflammatory cytokines in serum and spinal dorsal horn, and improves NPP in rats (Tian et al., 2021). Transforming growth factor-β1 is a well-known regulatory cytokine that participates in the response to cellular stress and is closely related to the function of the nervous system and the differentiation of MSCs. Treatment of human umbilical cord MSCs with transforming growth factor-β1 upregulate lncRNA UCA1 levels in exosomes, reducing NPP, microglial proliferation, and the production of inflammatory mediators *in vivo* and *in vitro* (Mou et al., 2023). Transplantation of adipose-derived MSCs transfected with fibroblast growth factor 1 significantly reduced the expression of TNF-α and GFAP in spinal cord tissue, and relieved the mechanical and thermal hypersensitivity responses induced by sciatic nerve injury (Forouzanfar et al., 2018). Other studies have shown that pro-enkephalin genetically engineered human bone marrow MSCs increase leukocyte enkephalin (L-EK) secretion, effectively alleviating NPP caused by chronic compressive injury in rats (Sun et al., 2017).

Furthermore, MSCs can play an immunoregulatory role by producing exosomes and play a role in alleviating NPP. Different studies have revealed that MSC-derived exosomes have been used to relieve NPP and other inflammatory diseases as a promising cell-free treatment strategy. Exosomes derived from human umbilical cord MSCs reduce mechanical allodynia and thermal hyperalgesia in rats with CFA (Hua et al., 2022). The analgesic functional effect of human umbilical cord MSC-derived exosomes reduces neuroinflammation by increasing the expression of autophagy-related proteins (LC3-II and beclin1) and inhibiting the activation of NLRP3 in the dorsal horn of the spinal cord (Hua et al., 2022). Intrathecal injection of MSC-derived vesicles significantly upregulated the expression of mRNA-99b-3p in spinal cord injury rats, activated autophagy levels in microglia, and alleviated mechanical allodynia caused by microglia activation (Gao et al., 2023). These studies have revealed that MSCs and their-derived exosomes can develop immunoregulatory and anti-inflammatory properties, thereby producing analgesic mechanisms.

## 5 Clinical trials and challenges of MSCs transplantation in pain treatment

Certain progress has been made in the application of MSCs in clinical trials to explore the safety, feasibility and therapeutic effect of treating different diseases, such as diabetes (Zang et al., 2022), heart failure (Bartolucci et al., 2017), acute non-arteritic optic neuropathy (Pastor et al., 2023) and rheumatoid arthritis (Wang et al., 2019). These clinical trials have confirmed the prospects of the application of MSCs as cell therapy. Given that MSCs play an analgesic role in basic pain research, similarly, MSCs have also achieved encouraging results in pain clinical trials. Some researchers have initially transitioned from basic research to clinical trials. Complicated pain in bony joints is a painful symptom experienced by most patients, causing dysfunction and discomfort to patients. MSCs transplantation improves joint function and relieves pain in patients. The phase lib clinical trial injected autologous fat-derived MSCs into the joint cavity of 12 patients with knee osteoarthritis and compared it with 12 knees injected with physiological saline (control group). After a 6-month follow-up, the primary outcome measures were the University of Western Ontario and McMaster University Osteoarthritis Index (WOMAC) scores and VAS scores, while secondary outcome measures included various clinical and radiological examinations and post-injection safety. Assessment of changes in cartilage defects using MRI. After MSCs transplantation, the VAS for knee pain was significantly reduced from 6.8 ± 0.6 to 3.4 ± 1.5, and pain symptoms were reduced. The patient’s activities of daily living, exercise and quality of life scores increased significantly (Lee et al., 2019). The arthritis patients achieved satisfactory functional improvement and pain relief, and no adverse events were caused during the 6-month follow-up (Lee et al., 2019). A further Phase III multicenter clinical trial reported that autologous adipose-derived MSCs were injected into the joint cavity of 161 patients with knee arthritis. After 6 months of follow-up, patients were assessed using the 100-mm Visual Analog Scale (VAS) and WOMAC, and the safety and changes in cartilage defects after MSCs transplantation were assessed using clinical, radiological and MRI. It was found that patients with osteoarthritis had significant pain relief and functional improvement, and no serious treatment-related adverse events were observed. MRI showed no significant differences in cartilage defects between the two groups at 6 months (Kim et al., 2023). Autologous human adipose-derived mesenchymal progenitor/stem cells were transplanted into the joint cavity of 53 patients with osteoarthritis. After 12 months of follow-up, pain and function were assessed using WOMAC score, visual analog score, and SF-36. The results showed that patients WOMAC, ASA and SF-36 scores all improved significantly, more patients had a 50% improvement in WOMAC, and more patients achieved a 70% improvement rate after 12 months. This suggests that patients were well tolerated and significantly improved knee joint function, pain, quality of life and cartilage regeneration (Lu et al., 2019). These studies reveal that intra-articular injection of MSCs can provide pain relief and functional improvement in patients with osteoarthritis, but long-term results are needed to determine the disease-improving effect of MSCs, such as the need for larger sample sizes, longer follow-up time, changes in bone structure, and the duration of the effect of intra-articular injection of MSCs on knee osteoarthritis.

A few clinical trials have shown the safety and efficacy of MSCs transplantation in the repair of spinal cord injuries and the treatment of concurrent NPP. A Phase 1/2a clinical trial reported the transplantation of Wharton jelly MSCs into 10 patients with chronic complete spinal cord injury. After 6 months of evaluation of clinical efficacy, the patients were evaluated using the American Spinal Injury Association Injury Scale motor and sensory scores, spasticity, neuropathic pain, electrical sensation, and pain threshold, lower limb motor evoked potentials (MEPs) and sensory evoked potentials (SEN), spinal cord independence measurements and the World Health Organization Quality of Life Bulletin, urodynamic studies and urine specific quality of life (Thomveen questionnaire), anorectal pressure measurements, assessment of intestinal dysfunction function (Rome III diagnostic questionnaire), and severity of fecal incontinence (Wexner score). The results showed that after MSCs transplantation, the acupuncture sensation in the skin area below the injury level was significantly improved. Other clinically relevant effects, such as the increased in bladder maximum capacity and compliance and decreased in bladder neurogenic ADHD and external sphincter dyssynergia, were only observed at the individual level. No changes were observed in motor function, spasticity, MEPs, SEPs, bowel function, quality of life, or independence indicators. This suggests that intrathecal transplantation of human umbilical cord derived WJ-MSCs is a safe intervention that can improve sensory changes in segments adjacent to the injury site (Albu et al., 2021). A phase II clinical trial showed intradural injection of MSCs into 11 patients, and the safety was analyzed after a 10-month follow-up. Patients were evaluated using clinical scales, urodynamics, neurophysiology and neuroimaging, and it was found that MSCs treatment was well tolerated and no adverse events related to MSCs administration occurred. Regardless of the degree of injury, age, or time after spinal cord injury, patients showed varying clinical improvements in sensitivity, exercise ability, spasticity, NPP, sexual function or sphincter dysfunction. Three patients originally classified as ASIA A, B and C were changed to ASIA B, C and D respectively. Neurophysiology showed improvement in somatosensory or motor evoked potentials in 55.5% of patients, and improvement in autonomous muscle contraction and active muscle reinnervation in lesions in 44.4% of patients (Vaquero et al., 2018a). This also suggests that MSCs therapy is safe and has shown effective therapeutic effects in SCI patients, mainly in terms of sphincter dysfunction, NPP relief and sensitivity recovery. Another clinical trial reported that 10 patients diagnosed with incomplete SCI received autologous bone marrow MSCs transplantation. After 12 months of follow-up, patients were evaluated through urodynamics, neurophysiology and neuroimaging, and the results showed that the patient’s sensitivity and motor function showed some improvement. Four patients experienced NPP before treatment; pain disappeared in two patients and pain decreased in another patient. Nine patients showed sub-lesion electromyography, indicating active muscle reinnervation (Vaquero et al., 2017). It was further found that the mean values of serum midbrain derived neurotrophic factor, glial derived neurotrophic factor, coriander neurotrophic factor, and neurotrophic factors 3/4 in patients after MSCs transplantation increased slightly compared with the basic level, but there was no statistically significant difference (Vaquero et al., 2017). This suggests that MSCs transplantation is a well-tolerated procedure that can achieve progressive and significant improvements in quality of life in patients with incomplete SCI. Another Phase 2 clinical trial reported that MSCs were transplanted into six paraplegic patients with syringomyelia. After 6 months of follow-up, treatment effects were assessed using clinical scales, urodynamics, neurophysiology, MRI, and anorectal pressure measurements. Anorectal pressure measurements improved in four patients. Four patients improved in neurophysiological studies, and three patients showed signs of improvement in evoked potentials. In the American Spinal Injury Society (ASIA) assessment, sensitivity improved in only two patients, but clinical improvements in neurogenic bowel dysfunction were observed in four patients. Three patients experienced NPP before treatment, but the pain decreased or disappeared completely after treatment. Only two adverse events occurred and were not related to cell therapy (Vaquero et al., 2018b) (Table 1).

**TABLE 1:** Clinical trials of MSCs in pain treatment.

Cell source	Pain type	Cell dose	Transplantation method	Follow-up time	Evaluation method	Therapeutic effect	References
Autologous adipose-derived MSCs	Knee osteoarthritis	1 × 10^8^ cells of MSCs in 3 mL	Intra-articular injection of	6 months	WOMAC score and VAS	Patients with knee osteoarthritis experienced satisfactory functional improvement and pain relief and caused no adverse events during the 6-month follow-up	Lee et al. (2019)
Autologous adipose-derived MSCs	Knee osteoarthritis	13 108ADMSCs; normal saline, 2.1 mL; autol ogous serum, 0.9 mL	Intra-articular injection	6 months	100-mm VAS and WOMAC sore	Patients with osteoarthritis experienced significant pain relief and functional improvement, and no serious treatment-related adverse events were observed	Kim et al. (2023)
Human adipose-derived MSCs	Knee osteoarthritis	5 × 10^7^	Intra-articular injection	12 months	WOMAC score, VAS and SF-36 assessment	The patient tolerated the MSCs transplantation well, and the patient’s joint function, pain, quality of life and cartilage regeneration significantly improved	Lu et al. (2019)
Wharton jelly mesenchymal stromal cells	(T3-11) Patients with chronic complete SCI	10 × 10^6^ cells	intrathecal grafting	6 months	(American Spinal Injury Association impairment scale motor and sensory score, spasticity, neuropathic pain, electrical perception and pain thresholds), lower limb motor evoked potentials (MEPs) and sensory evoked potentials (SEPs)	Intrathecal transplantation of MSCs is considered safe and has no significant side effects. The acupuncture sensation in the skin area below the injury level improved significantly	Albu et al. (2021)
Autologous mesenchymal stromal cells	Chronic SCI patients	100 × 10^6^ cells	Intradural injection	10 months	ASIA, Clinical Scales and Neurophysiology	MSCs treatment was well tolerated by the patients and no adverse events related to MSCs administration occurred. Regardless of the patient’s degree of injury, age, or time after injury, patients showed varying clinical improvements in sensitivity, exercise ability, spasticity, spasticity, NPP, sexual function or sphincter dysfunction, but mainly in terms of sphincter dysfunction, NPP and recovery of sensitivity	Vaquero et al. (2018a)
Autologous bone marrow MSCs	Patients with incomplete SCI	30 × 10^6^ cells	Subarachnoid injection	12 months	VAS,Neurophysiology and neuroimaging	After MSCs transplantation, patient sensitivity and motor function showed some improvement. Sexual function improved in two of the eight male patients. Four patients experienced neuropathic pain before treatment; two of them had pain disappeared and the other patient had pain reduced	Vaquero et al. (2017)
Autologous mesenchymal stromal cells	Paraplegic patient with syringomyelia	300 million MSCs	Intrathecal grafting	6 months	ASIA/Clinical Scales and Neurophysiology	MSCs transplantation reduced syringomyelia. Three patients experienced neuropathic pain before treatment, which reduced or disappeared completely after treatment. Only two adverse events occurred, which were not related to cell therapy	Vaquero et al. (2018b)

Although these clinical data reveal that MSCs transplantation has a functional effect on pain relief. However, the data and results obtained in these studies still lack enough direct evidence and better and reliable data to fully support the widespread development and application of MSCs in pain treatment. This is also related to some of the current problems and challenges in mesenchymal stem cell transplantation therapy. Question of the origin of MSCs. In clinical trials, most of the MSCs come from autologous adipose MSCs and bone marrow MSCs, which also poses challenges to cell origin in clinical trials. Although MSCs in clinical trials will not cause serious and long-term adverse reactions, including tumor malformations, the system for expanding and purifying MSCs *in vitro* is still imperfect and the gene instability generated during the culture process may lead to some side effects and other potential risks, which require careful consideration. This can be achieved by optimizing several aspects of MSCs isolation, propagation and vivo preparation, as well as the selection of doses used and individual dose adjustments for specific patients. Given that MSCs can produce exosomes, they also have a functional role in alleviating pain. Therefore, cell-free MSC-derived exosomes may be of interest in the field of tissue repair and pain relief because they do not have such potential side effects after administration. Moreover, the use of different doses of MSCs, the selection of transplant times, and the transplantation methods (including venous transplantation, intrathecal transplantation, or local transplantation) can lead to differences in treatment outcomes. For example, venous transplantation of MSCs may be difficult to colonize the injured site, which may lead to a decrease in the number of transplanted cells and cell survival, a decrease in the number of cells required for treatment and insignificant treatment effect, which may lead to differences and side effects in treatment effectiveness and safety. However, there are currently no standards to guide and implement completion, which is also an important factor hindering the widespread development of clinical trials. In addition, although MSCs have achieved encouraging results in clinical pain relief, more clinical trials and sufficient direct evidence are needed to support the safety, feasibility and effectiveness of its transplant treatment. Furthermore, more basic research and animal models are needed to explore the specific and detailed mechanisms of MSCs for alleviating pain *in vivo*. Because there are differences in physiology and development between humans and animals, the results obtained in animal models cannot be directly quoted into clinical trials, and because of fully understanding and controlling the biological characteristics of MSCs transplanted into the body and the favorable factors that produce analgesia. The standards and evaluation system for establishing animal models of NPP should also be better improved. In addition, we should also understand the differences in pain relief between MSCs and commonly used analgesics (such as opioids and non-opioids), maintenance time, and effect, as well as the interaction between MSCs and analgesics, including the therapeutic effect of combination use, and understand whether the combination of MSCs on the basis of analgesics application treatment can enhance the therapeutic effect of analgesics and the potential to reduce the dose and side effects of analgesics. Therefore, more preclinical and animal research are needed to understand the detailed molecular mechanisms and functional characteristics of MSCs in pain relief, so as to upgrade from basics to clinical practice to optimize and control the favorable therapeutic characteristics of MSCs, including their immune activity, anti-inflammatory properties, neurotrophic factor secretion, to maximize benefits. Therefore, intuitive results cannot be obtained directly from animal studies and can be directly extended to clinical trials (there are differences between animals and humans), and more research is needed to support and carry out.

## 6 Conclusion

The analgesic functions of MSCs are reflected in their promotion of angiogenesis, axon regeneration and myelination, neuron differentiation, production and secretion of neurotrophic factors, and immunomodulatory and anti-inflammatory properties. Moreover, MSC-derived exosomes have the functional characteristics of mother cells and can play a functional role in inhibiting pain progression by promoting cell proliferation, regulating inflammatory responses, reducing cell death, promoting axon regeneration, and promoting angiogenesis. These functions provide an important guarantee for pain relief. Significant progress has been made in the analgesic effect of MSCs in preclinical studies and animal models. These results have encouraged people to explore more in-depth and long-term applications of cell therapy. In addition, MSCs have also achieved some exciting results in clinical trials for pain relief, suggesting the safety and feasibility of their transplantation. As noted earlier, differences in their therapeutic effectiveness have also prevented their clinical trials. In addition, other issues such as variability in patient response, assessment of gender differences, and regulatory barriers are also important factors affecting the impact of MSCs on pain treatment. The widespread development of this is also related to the many problems and challenges existing in current MSCs therapy. Therefore, more basic research and pre-clinical trials are needed to explore and solve these current problems. Despite these problems that need to be solved in the future, MSCs have great potential and application value in pain relief.
